# Determination of the Duplicated *CYP2D6* Allele Using Real-Time PCR Signal: An Alternative Approach

**DOI:** 10.3390/jpm13060883

**Published:** 2023-05-24

**Authors:** Mazen A. Atiq, Sandra E. Peterson, Loralie J. Langman, Linnea M. Baudhuin, John L. Black, Ann M. Moyer

**Affiliations:** Department of Laboratory Medicine and Pathology, Mayo Clinic, 200 1st Street Southwest, Rochester, MN 55905, USA; atiq.mazen@mayo.edu (M.A.A.);

**Keywords:** *CYP2D6*, real-time PCR, Sanger sequencing, pharmacogenomics, duplications, copy number variation, targeted genotyping, long-range PCR

## Abstract

*CYP2D6* duplication has important pharmacogenomic implications. Reflex testing with long-range PCR (LR-PCR) can resolve the genotype when a duplication and alleles with differing activity scores are detected. We evaluated whether visual inspection of plots from real-time-PCR-based targeted genotyping with copy number variation (CNV) detection could reliably determine the duplicated *CYP2D6* allele. Six reviewers evaluated QuantStudio OpenArray *CYP2D6* genotyping results and the TaqMan Genotyper plots for seventy-three well-characterized cases with three copies of *CYP2D6* and two different alleles. Reviewers blinded to the final genotype visually assessed the plots to determine the duplicated allele or opt for reflex sequencing. Reviewers achieved 100% accuracy for cases with three *CYP2D6* copies that they opted to report. Reviewers did not request reflex sequencing in 49–67 (67–92%) cases (and correctly identified the duplicated allele in each case); all remaining cases (6–24) were marked by at least one reviewer for reflex sequencing. In most cases with three copies of *CYP2D6*, the duplicated allele can be determined using a combination of targeted genotyping using real-time PCR with CNV detection without need for reflex sequencing. In ambiguous cases and those with >3 copies, LR-PCR and Sanger sequencing may still be necessary for determination of the duplicated allele.

## 1. Introduction

The Cytochrome P450 (CYP) superfamily of enzymes are involved in the metabolism of a range of xenobiotics and their evolution parallels the need for host protection against environmental and food-produced toxins [1,2]. One critically important member is the CYP2D6 hepatic enzyme owing to its involvement in the metabolism of a vast array of commonly used drugs (approximately 20–25%) for diverse conditions—neuropsychiatric disorders, neoplasia, analgesia, antiemesis [3,4,5,6,7,8,9]. Particularly in psychiatry, the clinical relevance of this enzyme is highlighted by the many psychotropic medications (including antidepressants and neuroleptics) it metabolizes [10,11]. All this serves to underscore the need for accurate *CYP2D6* genotype-to-phenotype translation. However, with over 170 allelic variants including complex rearrangements, *CYP2D6* analysis is not straightforward, and accurate determination of the genotype can be challenging, particularly in the setting of structural variation [12,13,14,15,16,17]. *CYP2D6* copy number variations (CNVs), including full gene deletions and duplications, as well as hybrid alleles, are common, with a prevalence exceeding 12% in the United States population and, depending on the specific allele duplicated, can have important consequences for phenotype assignment [12,18,19,20,21]. Indeed, the inability to determine which allele is duplicated for heterozygous genotypes was a key challenge identified from a multi-site investigation of early *CYP2D6* clinical adopters, resulting in ambiguous phenotype assignment [22]. Similarly, proficiency survey testing, which assesses genotype and phenotype concordance between laboratories, found 53% of laboratories indicating an inability to distinguish a hybrid allele from a full gene duplication [23]. A general consensus from such studies is that a process to accurately determine the genotype and assign the phenotype must be in place in order to have appropriate reporting and downstream electronic clinical decision support.

In the clinical laboratory, long-range PCR to isolate the duplicated allele, coupled with either Sanger sequencing or genotyping of the long-range amplicon, can be employed as a gold-standard for definitive *CYP2D6* genotype determination when CNVs are present. However, many laboratories do not have the resources and/or expertise to incorporate this methodology into their workflows and instead often report the phenotype as “indeterminate”. While these methods are accurate, the major limitations to implementing them include that this methodology is labor-intensive, requires additional reagents and set-ups which increases turn-around-time (TAT), cannot reliably be performed on all specimen types (e.g., saliva, where the quantity and concentration are often too low for routine testing plus long-range PCR on one specimen), and requires specific expertise to interpret the results. Pyrosequencing has also been used for this purpose but can be challenging to interpret and is not widely used by clinical laboratories, so it would likely require additional instrumentation and an additional workflow to implement. Alternate methodologies that obviate the need for pyrosequencing or long-range PCR, such as analyzing signal ratios from mass-spectrometry-based techniques for single nucleotide variant (SNV) detection in conjunction with CNV information, have been described but their clinical implementation is hitherto unknown [12,24]. To minimize the use of a cumbersome methodology (long-range PCR followed by Sanger or genotyping) to determine *CYP2D6* genotype and predicted phenotype, we explored a more efficient alternate approach using plots generated using real-time-PCR-based SNV genotyping in samples with *CYP2D6* CNVs. We compared this method in a set of clinical cases to results generated using long-range PCR followed by Sanger sequencing to evaluate the feasibility and accuracy of this alternate approach in determining the duplicated allele and resolving the *CYP2D6* genotype and phenotype.

## 2. Materials and Methods

**Figure 1:**
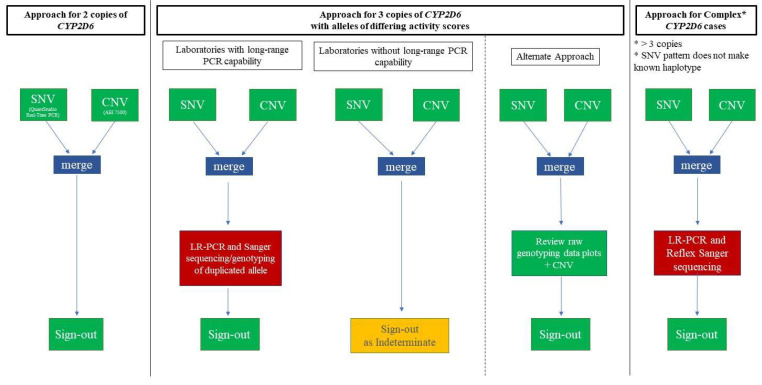
The typical *CYP2D6*-testing workflow incorporating a combination of single nucleotide variant (SNV) and copy number variation (CNV) detection (left portion of figure). Long-range PCR followed by sequencing or genotyping is used by some laboratories to report out genotypes when a duplication is present (second from left), while other laboratories report an indeterminate result (middle). An alternative approach uses only SNV (including the raw data) and CNV data to make a call for most cases (second from right). Complex cases may require LR-PCR and sequencing (right portion of figure).

The current clinical workflows are depicted in Figure 1 along with the alternative approach. Our laboratory’s typical workflow for cases with 3 copies of *CYP2D6* includes genotyping, CNV analysis, and long-range PCR to isolate the duplicated copy, followed by Sanger sequencing (corresponding to “Laboratories with Long-Range PCR Capabilities” in Figure 1). For this study, the current approach is compared to the “Alternate Approach” (Figure 1) that includes reviewing the signals from the raw genotyping data. Some complex cases may still require additional work-up with Sanger sequencing (see “Approach for Complex *CYP2D6* Cases” in Figure 1). For completeness, Figure 1 also depicts the pathway for cases that do not have a duplication, which are tested using only a combination of genotyping and CNV analysis prior to resulting. Finally, “Laboratories without Long-Range PCR Capabilities” are shown in Figure 1 with an “indeterminate” result in the setting of duplication; these laboratories may be able to implement the method described here.

In this study, 73 cases with 3 copies of *CYP2D6* were used to validate the alternate method (e.g., review of the raw genotyping data signals plus CNV data) for clinical use. In addition, the alternate method was compared with our standard approach for 11 cases with hybrid alleles and/or more than 3 copies of *CYP2D6*, and 3 cases with ambiguous CNV results (e.g., unclear if there were 2 or 3 copies present) for a total of 87 cases evaluated using both methods.

### 2.1. Assay Specifications

**Figure 2:**
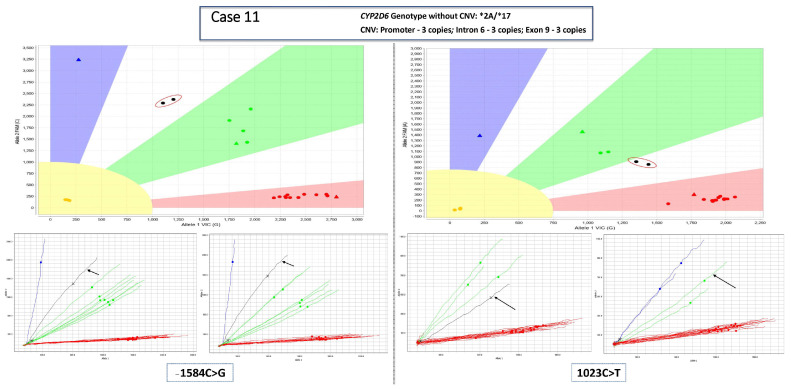
Example case presented to reviewers. Patient data (run in duplicate) are circled in red on each TaqMan Genotyper plot (top images). The two SNV assays shown are c.−1584C>G (pictured on the left and part of the *2A allele) and c.320C>T (legacy nomenclature: 1023C>T; pictured on the right and part of the *17 allele). *CYP2D6* is a reverse-strand gene, and the nucleotides indicated in the figure axes are relative to the genomic sequence (i.e., reverse complement of the coding strand). Colored triangles represent control samples with 2 copies of *CYP2D6* from prior runs. Colored circles represent patient samples run concurrently that have 2 copies of *CYP2D6*. The regions with blue and red shading correspond to location where homozygous reference or homozygous variant samples are expected, while the green shading corresponds to the region of the plot where heterozygous variant samples are expected. The bottom four diagrams represent sample trajectory traces (the black arrows point to the patient’s traces) used in the clustering algorithms of the Genotyper software. The blue and red traces correspond to homozygous reference or variant samples, while the green traces correspond to heterozygous samples. When the trace is black, it indicates that the software was unable to determine the genotype for that sample. Of note, the trajectory traces show all patients on the run, while the Genotyper plots only include those for whom *CYP2D6* will be reported.

**Figure 3:**
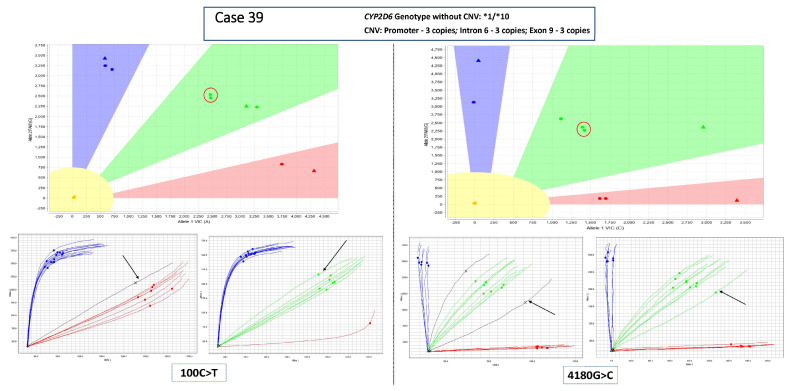
TaqMan Genotyper real-time PCR data plots for the *CYP2D6* c.100C>T (left) and c.1457G>C (legacy nomenclature 4180G>C, right) variants in a patient sample (run in duplicate and circled in red) with a *1×2/*10 genotype. The heterozygous zone (green shading in top figures) is wide due to lot-to-lot variability in signal, and there are few samples for comparison, which may make prediction of the duplicated allele challenging. The regions with blue and red shading correspond to location where homozygous reference or homozygous variant samples are expected. *CYP2D6* is a reverse-strand gene, and the nucleotides indicated in the figure axes are relative to the genomic sequence (i.e., reverse complement of the coding strand). Colored triangles represent control samples with 2 copies of *CYP2D6* from prior runs. Colored circles represent patient samples run concurrently that have 2 copies of *CYP2D6*. The bottom four diagrams represent sample trajectory traces (black arrows indicate the traces corresponding to this patient) used in the clustering algorithms of the Genotyper software. The blue and red traces correspond to homozygous reference or variant samples, while the green traces correspond to heterozygous samples. When the trace is black, it indicates that the software was unable to determine the genotype for that sample. Of note, the trajectory traces show all patients on the run, while the Genotyper plots only include those for whom *CYP2D6* will be reported.

All clinical samples were run in duplicate on the QuantStudio 12K Flex Real-Time PCR System (Thermo Fisher Scientific, Waltham, MA, USA) instrument using OpenArray technology and employing TaqMan chemistry with associated software version (1.2.2), TaqMan Genotyper software version 1.3 (Thermo Fisher Scientific, Waltham, MA, USA), and GINger version 1.0 (Mayo Clinic, Rochester, MN, USA). The results were used to generate qualitative SNV genotype calls, visualized using a classification scheme in the TaqMan Genotyper software. The classification scheme includes a pre-defined zone determining where samples (homozygous reference, heterozygous, or homozygous variant) are expected to fall based on samples run during development and test verification. The VIC and FAM fluorescent signals may vary between array lots, and the classification scheme zones are adjusted accordingly based on results of control samples with known genotypes. Although our laboratory typically analyzes data using the classification schemes, other laboratories using the same platform may use the clustering algorithm and review traces of the path each sample takes across the PCR cycles to the end point (i.e., sample trajectory). Images of this approach are included in the Figure 2 and Figure 3 for comparison.

The CNV assay was performed in duplicate on the ABI 7500 and/or ViiA 7 real-time instruments (Thermo Fisher Scientific, Waltham, MA, USA) using CopyCaller v.2.0 software (Thermo Fisher Scientific, Waltham, MA, USA) and interrogated three positions (promoter, intron 6, exon 9) within the *CYP2D6* locus. The results were then normalized to the *RPPH1* control gene.

As a comparator, DNA was first amplified using long-range PCR with primers designed to specifically amplify the downstream allele in the presence of a *CYP2D6* duplication (please see reaction C in Kramer WE, et al. for primer sequences) [21,25]. The long-range PCR products were then subjected to Sanger sequencing on the ABI 3730 xl DNA Analyzer (Thermo Fisher Scientific, Waltham, MA, USA) and data were analyzed using Mutation Surveyor version 4.0.9 (Soft Genetics, State College, PA, USA). Testing was performed in the clinical Personalized Genomics/Molecular Technologies laboratory of the Department of Laboratory Medicine and Pathology at Mayo Clinic, Rochester, MN, USA.

### 2.2. CYP2D6-Duplicated-Genotype Determination Exercise

Six reviewers with varying degrees of clinical experience (four laboratory directors, one clinical pathology resident, and one senior development technologist) visually inspected the raw data plots generated using real-time-PCR-based genotyping (on the classification scheme template used clinically in our laboratory) in combination with CNV data. Although not used in our laboratory, the sample trajectory traces used by some laboratories along with the clustering algorithms in the Genotyper software are also provided. The main dataset included 73 clinical cases (from clinical testing performed between November 2017 and September 2021) with a full-gene *CYP2D6* duplication (i.e., 3 copies) and alleles considered to have differing activity scores at the time of clinical testing. In addition, an exploratory dataset of 11 cases with hybrid alleles and/or more than 3 copies of *CYP2D6* were evaluated by four reviewers to test the method, although without the goal of clinical implementation at this time. Finally, three cases with ambiguous CNV results with the signal falling between 2 and 3 copies (i.e., “2.5 copies”) of the promoter, intron 6, and exon 9 were also evaluated. In addition to evaluation of the raw data using the alternate method described here and long-range PCR to isolate the duplicated allele, sequencing of all copies of *CYP2D6* present and the control gene, *RPPH1*, was performed.

For all cases, each reviewer was provided with (i) *CYP2D6* genotyping results, (ii) the CNV result for the promoter, intron 6, and exon 9 probes, and (iii) screen shots of the TaqMan Genotyper software plot for each heterozygous position (Figure 2). Reviewers visually assessed the location of the duplicates of each sample, which represents the relative VIC and FAM signals, on the TaqMan Genotyper plots relative to the position of the classification scheme zones and the reference, heterozygous, and homozygous variant clusters. Then the reviewers chose to either (1) indicate which allele was duplicated or (2) opt for follow-up sequencing if the reviewer deemed the results ambiguous and would not be comfortable calling the duplicated allele. Reviewers were blinded to the final clinical results when evaluating the cases. Results from this exercise were compared to the clinical results, which included genotype determined previously by integrating the targeted genotyping and CNV result with the sequencing data of the duplicated allele for each case. For the data tables in this study, all genotype-to-phenotype translations were standardized according to recent Clinical Pharmacogenetics Implementation Consortium (CPIC) and Dutch Pharmacogenetics Working Group (DPWG) consensus recommendations [26].

### 2.3. Statistical Analysis

Accuracy, inter-rater/reviewer reliability (inter-RR), and intra-rater/reviewer reliability (intra-RR) were evaluated. Accuracy was calculated by dividing the number of cases in which a reviewer correctly identified the duplicated allele by the total number of cases the reviewer attempted. When follow-up sequencing was requested, the case was excluded from the calculation. Inter-RR in predicting the correct vs. incorrect genotype was determined through the derivation of Cohen’s Kappa statistic for each rater pair for each case followed by the arithmetic mean of these estimates to provide an overall index of agreement [27]. For secondary analysis, inter-RR in choosing to report a case based on only SNV and CNV data vs. requesting sequencing was also calculated. Similarly, Cohen’s Kappa was determined to assess intra-RR by having reviewers repeat the exercise with the original cohort of cases, with the exception that case sequence arrangement differed from the original exercise. Cohen’s Kappa statistic can range from −1 to +1, where 0 represents the amount of agreement that can be expected from random chance, and 1 represents perfect agreement between the raters [28].

## 3. Results

The samples included in the study are shown in Table 1, which includes the genotype and phenotype as determined using the current clinical method (i.e., follow-up long-range PCR and Sanger sequencing), as well as the corresponding activity score and number of heterozygous SNVs available for review for each case. Briefly, in the main dataset (73 cases with 3 copies of the full *CYP2D6* gene), 30 different genotypes were evaluated with *1/*4×2 being the most common (n = 11 cases). Phenotypes ranged from intermediate to ultrarapid metabolizer with activity scores ranging from 0.5 to 3. The number of heterozygous SNVs for a given case ranged from one to five (median three). Among the 11 cases with hybrid alleles and/or more than 3 copies of *CYP2D6* in the exploratory dataset, the number of heterozygous SNVs for a given case ranged from 2 to 4 (median 3.5).

### 3.1. Main Dataset—Cases with 3 Copies of CYP2D6

The reviewers requested sequencing follow-up in 6–26 of the 73 cases (Table 1). In cases for which sequencing follow-up was not requested, the duplicated allele was called correctly with 100% accuracy by the reviewers, and inter-RR yielded perfect agreement among reviewers (Cohen’s kappa: 1.0). Similarly, intra-RR indicated perfect agreement (Cohen’s kappa = 1.0). For exploratory purposes, inter-RR was also determined using the two categories of “Correct” and “Sequenced” which yielded a Cohen’s kappa statistic of 0.3.

The number of reviewers who requested sequencing for any given case is also included in Table 1 and ranged from 0 to 6 (mean 0.8). For a given case, sequencing was more likely to be required when fewer informative (heterozygous) SNVs were present—e.g., 100% of cases with one SNV (n = 10) required sequencing according to at least one reviewer (mean 1.9). Conversely, only 3–37% of cases with 3 SNVs (n = 29), which was the median number of informative SNVs per case across the cohort, required sequencing (a mean 0.65 of reviewers requested sequencing per case). Reviewers also requested sequencing in some cases when there were few samples on the run with heterozygous calls because this made it more difficult to evaluate the “shift” of the sample with the duplication (Figure 3).

### 3.2. Complex Cases—Including Hybrid Alleles and/or More Than three Copies of CYP2D6

A subset of cases included hybrid alleles and/or more than three copies of *CYP2D6* (Table 1). Due to their complexity, the laboratory team did not attempt to validate the alternate method for these samples at this time. Therefore, these samples were included for exploratory purposes only and accuracy and inter-RR were not calculated. Select cases where calls were attempted demonstrated the effectiveness of this approach as all reviewers correctly predicted the duplicated/multiplicated allele in addition to any hybrid alleles that may be present. In most cases with hybrid alleles and CNV results indicating more than three copies, the reviewers felt that long-range PCR plus sequencing would remain the most appropriate approach at present. Of note, this group of cases included three particularly challenging cases that cannot be resolved without sequencing. While full-gene *CYP2D6* duplications typically include multiple copies of the same allele, one of the cases that had four total *CYP2D6* copies appeared on targeted genotyping to be heterozygous for the *2A and *17 alleles. After long-range PCR to isolate the duplicated allele(s), sequencing surprisingly revealed heterozygous *2 and *2A alleles (only the c.−1584C>G was heterozygous in the traces). Although the reviewers identified that the *2A allele was duplicated rather than the *17, the *2 allele without the c.−1584C>G variant was not expected. Using the techniques in the clinical laboratory, it was not possible to phase the alleles and fully resolve the diplotype, and the case was reported as *2A×2/*2+*17 or *2A×2+*2/*17. Another case had a very high copy number of the promoter probe (estimated at 14 copies using the software, far exceeding the validated range of the CNV assay) along with three copies each of intron 6 and exon 9. The reviewers correctly identified the presence of two copies of *2A and only one copy of *4 but suspected the presence of one or more*68 alleles (or another *CYP2D6–CYP2D7* hybrid allele) based on the CNV result. Finally, one case could not be definitively resolved despite sequencing as it had high copy numbers for each probe (predicted as six copies each of the promoter and intron 6 and five copies of exon 9) all of which are above the validated range of the assay. The reviewers could predict that the *1 allele was duplicated; however, copy number estimation was not possible.

### 3.3. Cases with Ambiguous CYP2D6 CNV Results

**Figure 4:**
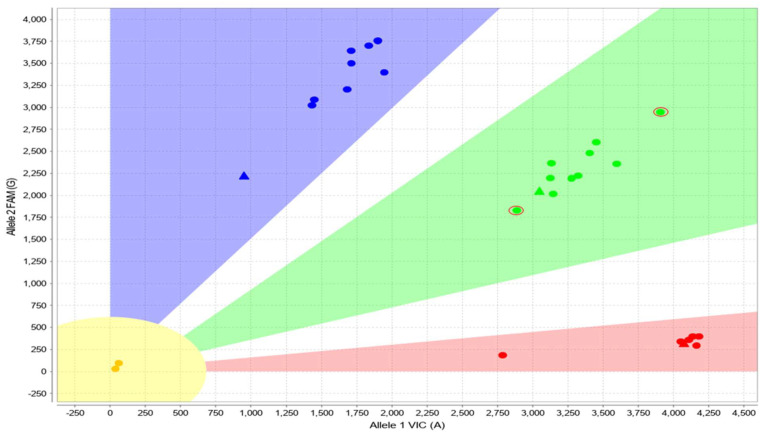
TaqMan Genotyper real-time PCR data plot for the *CYP2D6* c.886C>T variant (also known as 2850C>T in legacy nomenclature) present in a sample (run in duplicate, circled in red) with a *9/*35 genotype and “2.5” copies of *CYP2D6*, using CNV analysis. The patient sample (run in duplicate and circled in red) is located near the green triangle, where a typical heterozygous sample with two copies of *CYP2D6* is expected to fall, within the heterozygous zone (green shaded region). The red and blue shaded regions correspond to the zones where samples with homozygous reference and variant genotypes are expected. Colored circles represent patient samples with two copies that were run concurrently. *CYP2D6* is a reverse-strand gene, and the nucleotides indicated in the figure axes are relative to the genomic sequence (i.e., reverse complement of the coding strand).

Three cases were identified with ambiguous *CYP2D6* CNV results. These cases had a reproducible signal falling between 2 and 3 copies (e.g., “2.5 copies”) of *CYP2D6* at each of the 3 probe locations (promoter, intron 6, exon 9) using CNV analysis. After reflexing to long-range PCR/sequencing to attempt to identify the duplicated allele, no amplicon was visible on a check gel and the subsequent sequencing failed. Evaluation of these samples with the alternate approach suggested that a duplication may not be present. Specifically, the heterozygous SNVs in all three cases were not shifted on the TaqMan Genotyper plots (Figure 4). Sequencing of the control gene, *RPPH1* (NR_002312.1)*,* revealed the presence of a heterozygous n.47C>T (rs3093876) variant in two of the samples and a heterozygous n.74G>A (rs1225806543) variant in the third sample. These variants are expected to interfere with the copy number assay (personal communication with Thermo Fisher Scientific, September 2022), which could lead to decreased or no amplification of one copy of the control gene and incorrect *CYP2D6* copy number results.

## 4. Discussion

When a *CYP2D6* duplication is present, and two alleles with differing activity scores are identified, accurate determination of the duplicated allele is consequential for the corresponding phenotype assignment. This may impact medication recommendations for CYP2D6 substrates, including neuropsychiatric and pain-control medications such as tricyclic antidepressants, selective serotonin reuptake inhibitors (SSRIs), and codeine. Currently, many laboratories would report *CYP2D6* as “indeterminate” in this scenario. Other laboratories may perform reflex long-range PCR to isolate the duplicated allele, followed by either Sanger sequencing or genotyping of the amplicon to determine which allele is duplicated. In this study, a simple and cost-efficient approach is proposed—one that already aligns with the standard clinical workflow and is potentially feasible for all laboratories using QuantStudio instrumentation (regardless of whether they use a classification scheme or the clustering algorithm)—to determine the duplicated *CYP2D6* allele in the setting of heterozygous SNVs. In the presence of three copies of *CYP2D6,* accurate and reliable determination of the duplicated allele can be achieved in most straightforward cases with a combination of targeted genotyping using TaqMan-based real-time PCR and CNV detection across multiple loci, coupled with visual inspection of the raw data instead of expensive and time-consuming reflex long-range PCR followed by either Sanger sequencing or genotyping of the amplicon. This was demonstrated by a high degree of accuracy in making calls by six reviewers with varying degrees of experience. Where ambiguity remains, particularly for complex cases, long-range PCR (followed by either sequencing or genotyping of the product) may still be necessary to determine which allele is duplicated. Reduced *CYP2D6* reflex testing will likely lead to decreased turn-around-time (TAT) for patient results, cost savings, and fewer disruptions to the clinical schedule as long-range PCR is labor-intensive. Our laboratory did not validate *CYP2D6* long-range PCR and sequencing on saliva specimens due to insufficient yield and lower quality extractions (fragmented DNA). Using the alternate approach, the duplicated allele can be determined for saliva specimens, as well as blood specimens, resulting in more accurate genotype and phenotype reporting.

This approach has limitations. Although it is well-suited for cases with three copies of *CYP2D6* and multiple informative (heterozygous) SNVs, it may be challenging to apply in complex cases with hybrid alleles and/or more than three copies of *CYP2D6*. However, accurate calls may still be possible in select cases. For example, in cases with four copies of the promoter and three copies each of intron 6 and exon 9 and the presence of a *4 allele, most reviewers in this study were able to identify which allele was duplicated and predict the presence of a *68 hybrid allele. Where cases exhibit data scatter in the defined heterozygous zone (e.g., Figure 3), or a small number of heterozygous samples are on a run, ease of interpretability—and thus requests to sequence—may vary. To illustrate, two such cases with a *1×2/*10 diplotype had two (33%) and six (100%) reviewers request sequencing. The possibility of pooling samples from multiple runs into a larger dataset may allow for clearer cluster visualization but is unlikely to be feasible in a clinical setting where results must be returned in a timely manner. Although a request to sequence rather than the application of the alternate approach may have multiple underlying reasons, it is worth noting that the lowered inter-RR (0.3) when comparing “reported based on SNV and CNV data only” vs. “sequencing requested” may be attributed to the inherent structure of the exercise. Most reviewers attempted to complete review of all cases within a few sessions. In contrast, in the clinical laboratory setting, typically fewer than three cases with full gene duplication and heterozygous SNVs are addressed in any given clinical run. Thus, the mental fatigue from reviewing many cases in one sitting may have led to increased sequencing requests in this study exercise. Additionally, given that some cases were previously encountered by four reviewers (laboratory directors) during clinical reporting, recall bias cannot be excluded but is likely significantly mitigated by the wide timeframe (~4 years) from which cases in this cohort were selected.

Assay chemistry considerations may also pose challenges for this approach, particularly for clinical laboratories interrogating only one position during copy number evaluation. Laboratories utilizing CNV detection of only exon 9 will still not be able to definitively differentiate between a full copy of *CYP2D6* and a *CYP2D7–CYP2D6* (*13) hybrid allele, even if using this approach. Additionally, as recently described, employing only one control gene (*RPPH1*) in standard qPCR assays may also impact CNV results [29]. Interestingly, the approach proposed here could help in troubleshooting such ambiguous CNV results. When the method described here was applied to the three samples that had an ambiguous CNV result of “2.5” at all three loci, the TaqMan Genotyper plots for heterozygous SNVs revealed data points centered within the defined zones and not skewed, supporting the absence of a duplication. Similar to the study where inaccurate copy number assignments were described in the setting of *CFTR* and *SMN2* CNV testing due to sequence variants within the primer/probe binding sites of the control gene, *RPPH1*, in each of our cases a heterozygous variant was also identified in *RPPH1* [29]. In addition to using the method proposed here, use of a second control gene, such as *CYP2D8P*, could be helpful [30].

In addition to control gene variants, rare variants may be present in *CYP2D6*, given its polymorphic nature, and such variants may interfere with a primer or probe in the CNV assay, causing allelic drop out in the targeted genotyping assay, or may remain undetected using targeted genotyping methodologies [31,32,33,34,35]. In our laboratory, a set of long-range PCR reactions have been designed to isolate specific *CYP2D6* gene copies, which can be used to resolve these cases. When a rare variant is identified, it must be evaluated to determine whether it is reportable due to expected impact on the final phenotype [21,36,37,38]. Sequencing performed for these cases differs from the sequencing performed to identify the duplicated allele and will not be decreased by the method used here. Reliance on a single heterozygous SNV to make calls using the alternate approach can also prove problematic if allelic drop out skews the plot in favor of the non-duplicated allele. As evidenced by a *1×2/*41 case (not shown), an intronic variant (c.844−12G>A) close to the c.886C>T (legacy 2850C>T) variant present in the *41 allele caused drop out of the wild-type allele suggesting increased copies of the alternate allele (*41). In contrast, data for the c.985+39G>A (legacy 2988G>A) and the c.1457G>C (legacy 4180G>C) variants suggested duplication of the *1. Had additional SNVs not been available and considered, it would be easy to be misled into an incorrect call due to allelic drop-out or inefficient amplification of one allele in the setting of a rare variant.

Relevant alternative evolving methodologies exist that may ultimately replace the need for the approach described here and warrant mention [39,40]. Although most clinical laboratories are not yet using next-generation sequencing (NGS) for pharmacogenomics (PGx), bioinformatic tools have been developed to determine the *CYP2D6* genotype, which in some cases includes CNV, using NGS data [37,41,42,43,44,45,46,47,48]. These tools are rapidly emerging and may facilitate clinical implementation of PGx using NGS. However, these tools have limitations, particularly when applied to short-read NGS chemistry. A pediatric cohort study assessing concordance between whole-genome sequencing (WGS), whole-exome sequencing (WES), and targeted genotyping for 19 pharmacogenes generally found high concordance (>99%) among the various methodologies but discovered NGS issues specific to *CYP2D6* (e.g., inability to accurately genotype *2 and *4 alleles), and thus ultimately recommended reflexing to targeted genotyping for *CYP2D6* in a clinical setting [49]. However, the limitation of targeted genotyping with respect to missing clinically actionable variants—ones not included in the test design—needs recognition. A recent study evaluating the potential patient impact (n = 10,030) of targeted genotyping versus NGS in a range of pharmacogenes found underperformance of standard targeted genotyping panels by missing potentially clinically relevant variant/alleles in 28% (n = 2780) of cases [50]. *CYP2D6* was amongst the genes with the highest number of missed potentially significant variants (n = 103) that could result in phenotype misclassification using targeted genotyping compared to NGS approaches.

Long-read NGS is expected to solve some of the challenges of short-read NGS chemistry [40,51,52]. However, as with any sequencing-based approach, the discovery of rare or novel alleles may present challenges in reporting and predicting the metabolizer phenotype from the genotype. This issue may only be addressed with structured variant interpretation and nomenclature systems that can accommodate novel and rare variants [50]. In the presence of variants of uncertain significance, a phenotype range or use of a continuous activity score without formal translation to phenotype have been suggested and may particularly benefit the reporting of *CYP2D6* given its polymorphic nature, and the likely transition to sequencing (versus targeted genotyping) in the future [26,50,53]. Overall, NGS-based approaches may ultimately replace the approach to identifying the duplicated allele in targeted genotyping that is described here; however, given the challenges of NGS and interpreting/reporting rare variants, targeted genotyping platforms will likely be in clinical use for some time yet.

In summary, we present an alternative approach to Sanger sequencing to resolve the *CYP2D6* genotype and phenotype in the event of a duplication. This approach may be feasible for other clinical laboratories using real-time PCR and QuantStudio instruments for *CYP2D6* genotyping. This approach uses the standard genotyping and CNV data routinely generated; therefore, compared to follow-up Sanger sequencing which requires additional reagents and technologist time, this approach may lead to cost-savings, a shorter TAT for patient results, and may allow for determining the duplicated allele in additional specimen types that are not amenable to long-range PCR. Until NGS for pharmacogenetic testing becomes ubiquitous and replaces targeted genotyping, the approach proposed here can allow for more precise genotyping in the setting of a duplication and/or decrease the need for Sanger sequencing follow-up.

## Figures and Tables

**Table 1:** Process Improvement Study Samples.

Main Dataset: Cases with 3 Copies of *CYP2D6* (n = 73)						
Genotype	Total Cases		Phenotype	Activity Score	Number of Het SNVs	Number of Reviewers Who Requested Sequencing for Each Case †
*1/*4×2	11		Intermediate	1	3	0
						0
						0
						0
						2
						0
						1
						0
						0
						3
						1
*1×2/*4	7		Normal	2	3	1
						0
						0
						0
						1
						2
						0
*1×2/*41	6		Ultrarapid	2.5	3	1
						1
						0
						1
						1
						1
*1/*2×2	6		Ultrarapid	3	2	0
						0
						0
						0
						0
						0
*4×2/*17	5		Intermediate	0.5	4	0
						1
						1
						0
						0
*2A×2/*41	4		Ultrarapid	2.5	2	1
						2
						1
						4
*1×2/*9	4		Ultrarapid	2.5	1	1
						1
						1
						1
*2A×2/*4	3		Normal	2	4	0
						0
						0
*2A×2/*17	2		Ultrarapid	2.5	2	0
						0
*1/*41×2	2		Normal	2	3	1
						0
*2×2/*9	2		Ultrarapid	2.5	3	5
						3
*2×2/*2A	2		Ultrarapid	3	1	2
						2
*1×2/*10	2		Normal	2.25	2	2
						6
*4/*35×2	1		Normal	2	3	0
*27×2/*41	1		Ultrarapid	2.5	2	2
*9/*35×2	1		Ultrarapid	2.5	5	1
*4×2/*59	1		Intermediate	0.5	4	3
*4×2/*41	1		Intermediate	0.5	4	1
*4×2/*35	1		Intermediate	1	5	1
*2×2/*35	1		Ultrarapid	3	2	2
*2×2/*4	1		Normal	2	1	1
*2A×2/*29	1		Ultrarapid	2.5	2	2
*2A×2/*10	1		Normal	2.25	3	0
*2A×2/*9	1		Ultrarapid	2.5	4	0
*2A×2/*6	1		Normal	2	4	0
*2A/*4×2	1		Intermediate	1	4	0
*2/*4×2	1		Intermediate	1	3	1
*1×2/*6	1		Normal	2	1	1
*1×2/*3	1		Normal	2	1	1
*1/*2A×2	1		Ultrarapid	3	3	0
**Exploratory Data Set: Cases with Hybrid Alleles and/or >3 copies of *CYP2D6* (n = 11)**						
**Genotype**	**CNV §**	**Total Cases**	**Phenotype**	**Activity Score**	**Number of Het SNVs**	**Number of Reviewers Requesting Sequencing †**
*68+4/*41×2	4, 3, 3	2	Intermediate	1	4	1/4
						2/4
*1×2/*68+*4	4, 3, 3	2	Normal	2	3	1/4
						2/3
*2A×2/*68+*4	4, 3, 3	1	Normal	2	4	1/4
*1×N/*68+*4	6, 6, 5	1	Ultrarapid	>3	3	4/4
*13+*2A×2/*9	3, 4, 4	1	Ultrarapid	2.5	4	1/4
*1×2/*36+*10	4, 4, 3	1	Normal	2	2	4/4
*4×2/*29×2	4, 4, 4	1	Intermediate	0.5	4	4/4
*2A×2/*2+*17 or *2A×2+*2/*17	4, 4, 4	1	Ultrarapid	3.5	2	4/4
*2A×2/*4	14, 3, 3	1	Normal	2	4	4/4
**Ambiguous CNV Status between 2 and 3 (n = 3)**						
**Genotype**	**Total Cases**		**Phenotype**	**Activity Score**	**Number of Het SNVs**	
*1/*6	1		Intermediate	1	1	
*2A/*35	1		Normal	2	1	
*9/*35	1		Normal	1.5	5	

†: Out of 6 reviewers, unless otherwise specified. §: *CYP2D6* copy number corresponding to the promoter, intron 6, and exon 9 probes, respectively.

## Data Availability

Data available upon request.

## References

[1] Esteves F., Rueff J., Kranendonk M. (2021). The Central Role of Cytochrome P450 in Xenobiotic Metabolism-A Brief Review on a Fascinating Enzyme Family. J. Xenobiot..

[2] Fuselli S., de Filippo C., Mona S., Sistonen J., Fariselli P., Destro-Bisol G., Barbujani G., Bertorelle G., Sajantila A. (2010). Evolution of detoxifying systems: The role of environment and population history in shaping genetic diversity at human CYP2D6 locus. Pharm. Genom..

[3] Hoskins J.M., Carey L.A., McLeod H.L. (2009). CYP2D6 and tamoxifen: DNA matters in breast cancer. Nat. Rev. Cancer.

[4] Penas-Lledo E.M., Llerena A. (2014). CYP2D6 variation, behaviour and psychopathology: Implications for pharmacogenomics-guided clinical trials. Br. J. Clin. Pharmacol..

[5] Taylor C., Crosby I., Yip V., Maguire P., Pirmohamed M., Turner R.M. (2020). A Review of the Important Role of CYP2D6 in Pharmacogenomics. Genes.

[6] Zanger U.M., Schwab M. (2013). Cytochrome P450 enzymes in drug metabolism: Regulation of gene expression, enzyme activities, and impact of genetic variation. Pharmacol. Ther..

[7] Ingelman-Sundberg M. (2004). Pharmacogenetics of cytochrome P450 and its applications in drug therapy: The past, present and future. Trends Pharmacol. Sci..

[8] Saravanakumar A., Sadighi A., Ryu R., Akhlaghi F. (2019). Physicochemical Properties, Biotransformation, and Transport Pathways of Established and Newly Approved Medications: A Systematic Review of the Top 200 Most Prescribed Drugs vs. the FDA-Approved Drugs between 2005 and 2016. Clin. Pharm..

[9] CPIC CYP2D6 CPIC Guidelines. https://cpicpgx.org/gene/cyp2d6/.

[10] Bertilsson L., Dahl M.L., Dalen P., Al-Shurbaji A. (2002). Molecular genetics of CYP2D6: Clinical relevance with focus on psychotropic drugs. Br. J. Clin. Pharmacol..

[11] Thummler S., Dor E., David R., Leali G., Battista M., David A., Askenazy F., Verstuyft C. (2018). Pharmacoresistant Severe Mental Health Disorders in Children and Adolescents: Functional Abnormalities of Cytochrome P450 2D6. Front. Psychiatry.

[12] Del Tredici A.L., Malhotra A., Dedek M., Espin F., Roach D., Zhu G.D., Voland J., Moreno T.A. (2018). Frequency of CYP2D6 Alleles Including Structural Variants in the United States. Front. Pharmacol..

[13] Nofziger C., Paulmichl M. (2018). Accurately genotyping CYP2D6: Not for the faint of heart. Pharmacogenomics.

[14] Gaedigk A., Ingelman-Sundberg M., Miller N.A., Leeder J.S., Whirl-Carrillo M., Klein T.E., PharmVar Steering C. (2018). The Pharmacogene Variation (PharmVar) Consortium: Incorporation of the Human Cytochrome P450 (CYP) Allele Nomenclature Database. Clin. Pharmacol. Ther..

[15] Gaedigk A., Whirl-Carrillo M., Pratt V.M., Miller N.A., Klein T.E. (2020). PharmVar and the Landscape of Pharmacogenetic Resources. Clin. Pharmacol. Ther..

[16] Gaedigk A., Casey S.T., Whirl-Carrillo M., Miller N.A., Klein T.E. (2021). Pharmacogene Variation Consortium: A Global Resource and Repository for Pharmacogene Variation. Clin. Pharmacol. Ther..

[17] Pharmacogene Variation Consortium CYP2D6. https://www.pharmvar.org/gene/CYP2D6.

[18] Jarvis J.P., Peter A.P., Shaman J.A. (2019). Consequences of CYP2D6 Copy-Number Variation for Pharmacogenomics in Psychiatry. Front. Psychiatry.

[19] Beoris M., Amos Wilson J., Garces J.A., Lukowiak A.A. (2016). CYP2D6 copy number distribution in the US population. Pharm. Genom..

[20] Pratt V.M., Cavallari L.H., Del Tredici A.L., Gaedigk A., Hachad H., Ji Y., Kalman L.V., Ly R.C., Moyer A.M., Scott S.A. (2021). Recommendations for Clinical CYP2D6 Genotyping Allele Selection: A Joint Consensus Recommendation of the Association for Molecular Pathology, College of American Pathologists, Dutch Pharmacogenetics Working Group of the Royal Dutch Pharmacists Association, and the European Society for Pharmacogenomics and Personalized Therapy. J. Mol. Diagn..

[21] Black J.L., Walker D.L., O'Kane D.J., Harmandayan M. (2012). Frequency of undetected CYP2D6 hybrid genes in clinical samples: Impact on phenotype prediction. Drug Metab. Dispos..

[22] Cavallari L.H., Van Driest S.L., Prows C.A., Bishop J.R., Limdi N.A., Pratt V.M., Ramsey L.B., Smith D.M., Tuteja S., Duong B.Q. (2019). Multi-site investigation of strategies for the clinical implementation of CYP2D6 genotyping to guide drug prescribing. Genet. Med..

[23] Moyer A.M., McMillin G.A., Long T.A., Gandhi M.J., Mao R., Smock K.J., Halley J.G., Weck K.E. (2020). Genotype and Phenotype Concordance for Pharmacogenetic Tests through Proficiency Survey Testing. Arch. Pathol. Lab. Med..

[24] Carvalho Henriques B., Buchner A., Hu X., Wang Y., Yavorskyy V., Wallace K., Dong R., Martens K., Carr M.S., Behroozi Asl B. (2021). Methodology for clinical genotyping of CYP2D6 and CYP2C19. Transl. Psychiatry.

[25] Kramer W.E., Walker D.L., O'Kane D.J., Mrazek D.A., Fisher P.K., Dukek B.A., Bruflat J.K., Black J.L. (2009). CYP2D6: Novel genomic structures and alleles. Pharm. Genom..

[26] Caudle K.E., Sangkuhl K., Whirl-Carrillo M., Swen J.J., Haidar C.E., Klein T.E., Gammal R.S., Relling M.V., Scott S.A., Hertz D.L. (2020). Standardizing CYP2D6 Genotype to Phenotype Translation: Consensus Recommendations from the Clinical Pharmacogenetics Implementation Consortium and Dutch Pharmacogenetics Working Group. Clin. Transl. Sci..

[27] Hallgren K.A. (2012). Computing Inter-Rater Reliability for Observational Data: An Overview and Tutorial. Tutor. Quant. Methods Psychol..

[28] McHugh M.L. (2012). Interrater reliability: The kappa statistic. Biochem. Med..

[29] Sicko R.J., Romitti P.A., Browne M.L., Brody L.C., Stevens C.F., Mills J.L., Caggana M., Kay D.M. (2022). Rare Variants in RPPH1 Real-Time Quantitative PCR Control Assay Binding Sites Result in Incorrect Copy Number Calls. J. Mol. Diagn..

[30] Larsen J.B., Jorgensen S. (2022). Simple and Robust Detection of CYP2D6 Gene Deletions and Duplications Using CYP2D8P as Reference. Pharmaceuticals.

[31] Riffel A.K., Dehghani M., Hartshorne T., Floyd K.C., Leeder J.S., Rosenblatt K.P., Gaedigk A. (2015). CYP2D7 Sequence Variation Interferes with TaqMan CYP2D6 (*) 15 and (*) 35 Genotyping. Front. Pharmacol..

[32] Gaedigk A., Riffel A.K., Leeder J.S. (2015). CYP2D6 Haplotype Determination Using Long Range Allele-Specific Amplification: Resolution of a Complex Genotype and a Discordant Genotype Involving the CYP2D6*59 Allele. J. Mol. Diagn..

[33] Rasmussen H.B., Werge T. (2011). Novel variant of CYP2D6*6 is undetected by a commonly used genotyping procedure. Pharmacol. Rep..

[34] Turner A.J., Aggarwal P., Boone E.C., Haidar C.E., Relling M.V., Derezinski A.D., Broeckel U., Gaedigk A. (2021). Identification of CYP2D6 Haplotypes that Interfere with Commonly Used Assays for Copy Number Variation Characterization. J. Mol. Diagn..

[35] Scantamburlo G., Tziolia K., Zopf M., Bernardinelli E., Soyal S.M., Civello D.A., Vanoni S., Dossena S., Patsch W., Patrinos G.P. (2017). Allele Drop out Conferred by a Frequent CYP2D6 Genetic Variation For Commonly Used CYP2D6*3 Genotyping Assays. Cell Physiol. Biochem..

[36] Zhou Y., Tremmel R., Schaeffeler E., Schwab M., Lauschke V.M. (2022). Challenges and opportunities associated with rare-variant pharmacogenomics. Trends Pharmacol. Sci..

[37] Wang L., Scherer S.E., Bielinski S.J., Muzny D.M., Jones L.A., Black J.L., Moyer A.M., Giri J., Sharp R.R., Matey E.T. (2022). Implementation of preemptive DNA sequence-based pharmacogenomics testing across a large academic medical center: The Mayo-Baylor RIGHT 10K Study. Genet. Med..

[38] Ingelman-Sundberg M., Mkrtchian S., Zhou Y., Lauschke V.M. (2018). Integrating rare genetic variants into pharmacogenetic drug response predictions. Hum. Genom..

[39] Yang Y., Botton M.R., Scott E.R., Scott S.A. (2017). Sequencing the CYP2D6 gene: From variant allele discovery to clinical pharmacogenetic testing. Pharmacogenomics.

[40] Liau Y., Maggo S., Miller A.L., Pearson J.F., Kennedy M.A., Cree S.L. (2019). Nanopore sequencing of the pharmacogene CYP2D6 allows simultaneous haplotyping and detection of duplications. Pharmacogenomics.

[41] Twist G.P., Gaedigk A., Miller N.A., Farrow E.G., Willig L.K., Dinwiddie D.L., Petrikin J.E., Soden S.E., Herd S., Gibson M. (2017). Erratum: Constellation: A tool for rapid, automated phenotype assignment of a highly polymorphic pharmacogene, CYP2D6, from whole-genome sequences. NPJ Genom. Med..

[42] Lee S.B., Wheeler M.M., Patterson K., McGee S., Dalton R., Woodahl E.L., Gaedigk A., Thummel K.E., Nickerson D.A. (2019). Stargazer: A software tool for calling star alleles from next-generation sequencing data using CYP2D6 as a model. Genet. Med..

[43] Qiao W., Wang J., Pullman B.S., Chen R., Yang Y., Scott S.A. (2017). The CYP2D6 VCF Translator. Pharm. J..

[44] Numanagic I., Malikic S., Ford M., Qin X., Toji L., Radovich M., Skaar T.C., Pratt V.M., Berger B., Scherer S. (2018). Allelic decomposition and exact genotyping of highly polymorphic and structurally variant genes. Nat. Commun..

[45] Twesigomwe D., Drogemoller B.I., Wright G.E.B., Siddiqui A., da Rocha J., Lombard Z., Hazelhurst S. (2021). StellarPGx: A Nextflow Pipeline for Calling Star Alleles in Cytochrome P450 Genes. Clin. Pharmacol. Ther..

[46] Chen X., Shen F., Gonzaludo N., Malhotra A., Rogert C., Taft R.J., Bentley D.R., Eberle M.A. (2021). Cyrius: Accurate CYP2D6 genotyping using whole-genome sequencing data. Pharm. J..

[47] Twesigomwe D., Wright G.E.B., Drogemoller B.I., da Rocha J., Lombard Z., Hazelhurst S. (2020). A systematic comparison of pharmacogene star allele calling bioinformatics algorithms: A focus on CYP2D6 genotyping. NPJ Genom. Med..

[48] Hari A., Zhou Q., Gonzaludo N., Harting J., Scott S.A., Qin X., Scherer S., Sahinalp S.C., Numanagic I. (2023). An efficient genotyper and star-allele caller for pharmacogenomics. Genome Res..

[49] Cohn I., Paton T.A., Marshall C.R., Basran R., Stavropoulos D.J., Ray P.N., Monfared N., Hayeems R.Z., Meyn M.S., Bowdin S. (2017). Genome sequencing as a platform for pharmacogenetic genotyping: A pediatric cohort study. NPJ Genom. Med..

[50] Lopes J.L., Harris K., Karow M.B., Peterson S.E., Kluge M.L., Kotzer K.E., Lopes G.S., Larson N.B., Bielinski S.J., Scherer S.E. (2022). Targeted Genotyping in Clinical Pharmacogenomics: What Is Missing?. J. Mol. Diagn..

[51] Ammar R., Paton T.A., Torti D., Shlien A., Bader G.D. (2015). Long read nanopore sequencing for detection of HLA and CYP2D6 variants and haplotypes. F1000Res.

[52] Qiao W., Yang Y., Sebra R., Mendiratta G., Gaedigk A., Desnick R.J., Scott S.A. (2016). Long-Read Single Molecule Real-Time Full Gene Sequencing of Cytochrome P450-2D6. Hum. Mutat..

[53] Van der Lee M., Allard W.G., Vossen R., Baak-Pablo R.F., Menafra R., Deiman B., Deenen M.J., Neven P., Johansson I., Gastaldello S. (2021). Toward predicting CYP2D6-mediated variable drug response from CYP2D6 gene sequencing data. Sci. Transl. Med..

